# CFTR modulators partially restore the epithelial interferome in *Aspergillus* infection to improve clinical outcome

**DOI:** 10.1016/j.ebiom.2026.106131

**Published:** 2026-01-31

**Authors:** Sarah L. Laverty, Imogen Felton, Michelle Casey, Benjamin Hopwood, Nicholas Simmonds, Darius Armstrong-James, Anand Shah, Peter Kelleher

**Affiliations:** a Imperial Fungal Science Network, Department of Infectious Disease, Imperial College London, UK; b Immunology of Infection, Department of Infectious Disease, Imperial College London, UK; c Royal Brompton and Harefield Hospitals, Guy's and St. Thomas' NHS Foundation Trust, London, UK; d National Heart and Lung Institute, Imperial College London & NIHR Biomedical Research Centre, UK; e Adult Cystic Fibrosis Centre, Beaumont Hospital, Dublin, Ireland

**Keywords:** Cystic fibrosis, Aspergillus, Interferon, Immunity, Neutrophil

## Abstract

**Background:**

The impact of CFTR modulator therapy on host immunity and outcomes in people with Cystic Fibrosis (CF)-related Aspergillus lung disease is poorly defined. We aimed to characterise fungal-relevant clinical outcomes post-CFTR modulators and assess effects on the Aspergillus-dependent Type I/III interferome.

**Methods:**

Biomarkers of Aspergillus-related lung disease (Aspergillus-specific IgE/IgG), anti-fungal and corticosteroid therapy were analysed in a retrospective cohort of people with CF pre and post Elexacaftor/Tezacaftor/Ivacaftor (ETI) modulator therapy. Homozygous *F508del* (CF) and CFTR TALEN-corrected bronchial epithelial cells (BECs) were challenged with *Aspergillus* conidia and hyphae in the presence or absence of ETI CFTR modulator therapy with bulk RNA transcriptomics and RT-PCR used to analyse Type I/III interferon genes. Effects of exogenous type I and III interferons on CF-neutrophil antifungal effector function was further characterised.

**Findings:**

CFTR modulator (ETI) therapy was associated with a significant reduction in *Aspergillus* biomarkers alongside use of corticosteroid and anti-fungal therapy. In vitro Aspergillus stimulation enriched the Type I/III interferome in CFTR-corrected BECs compared to CF BECs, with ETI therapy partially restoring type I/III interferon gene expression in CF BECs. Administration of exogenous IFNλ1 increased anti-fungal killing in CF neutrophils without increased reactive-oxygen species or neutrophil extracellular trap production.

**Interpretation:**

CFTR modulators have led to improved clinical outcomes in CF related *Aspergillus-*related lung disease potentially due to partial restoration of the host antifungal epithelial type I/III interferon response. Exogenous IFNλ1 further improved antifungal killing capacity of CF-neutrophils presenting a plausible future therapeutic strategy.

**Funding:**

This study was funded by the 10.13039/501100000292Cystic Fibrosis Trust (SRC015).

**Research in context**

**Evidence before this study**

Cystic fibrosis (CF) is an autosomal recessive systemic disease caused by a mutation in the cystic fibrosis transmembrane conductance regulator (CFTR) gene whereby individuals affected are susceptible to disease due to the ubiquitous mould *Aspergillus fumigatus*. Although outcomes for individuals with CF have significantly improved following the availability of CFTR modulator therapy, there is currently a paucity of data on the effects of modulator therapy on fungal-specific clinical outcomes and immune responses to *A. fumigatus* infection. Downregulation of both Type I and III interferons have been observed in response to bacterial and viral infection in the CF lung, however the role of the respiratory epithelium and specifically the effects of CFTR modulator on the anti-fungal epithelial immune response is poorly understood.

**Added value of this study**

In this study, we use a large retrospective cohort study to highlight the impact of CFTR modulator therapy with a reduction in *A. fumigatus* specific biomarkers and microbiological growth. To analyse the potential immune-dependant mechanism of this finding, we utilised an in vitro bronchial epithelial cell model to analyse the CFTR-dependent interferome following Aspergillus infection. We show a novel CFTR-dependent downregulation of the epithelial interferome in response to *A. fumigatus* stimulation with partial correction following CFTR modulator therapy. Exogenous administration of IFNλ1 in vitro further augmented the capacity of neutrophils isolated from individuals with CF to eliminate *A. fumigatus* spores while also reducing excessive potentially harmful pro-inflammatory immune responses.

**Implications of all the available evidence**

Through this study, we show that introduction of highly effective CFTR modulator therapy leads to improved *A. fumigatus* infection-related clinical outcomes in people with CF potentially related to a partial restoration of an anti-fungal epithelial type I and III interferon host defence response. Exogenous administration of IFNλ1further presents a potential novel immunotherapeutic target to improve outcomes of fungal infection in CF.

## Introduction

Cystic fibrosis (CF) is an autosomal recessive systemic disease caused by mutations in the cystic fibrosis transmembrane conductance regulator (CFTR) gene.^1^ The protein defect in CFTR leads to defective mucociliary clearance, chronic microbial colonisation, recurrent infection, reduction in microbial killing by phagocytic cells and neutrophilic inflammation.^2^ *A. fumigatus* is a ubiquitous fungus which causes lower airway infection and allergic inflammation in individuals with CF.^3^ It is associated with accelerated decline in lung function^4^ and treatment is complicated by high rates of anti-fungal resistance and toxicity.^5^ Although outcomes for people with CF have significantly improved following the availability of triple CFTR modulator therapy (ETI), there is currently a paucity of data on the effects of modulator therapy on fungal-specific clinical outcomes and immune responses to *A. fumigatus* infection.^6^

Host defence against *Aspergillus* involves a co-ordinated cytokine response between respiratory epithelial cells, tissue resident macrophages and myeloid dendritic cells (DC), inflammatory monocytes, plasmacytoid DC and neutrophils recruited from blood.^7^ Type I IFN secreted by inflammatory CC chemokine receptor 2+ (CRR2+) monocytes promote Type III interferon mediated neutrophil *A. fumigatus* killing.^8^^,^^9^ Downregulation of both Type I and III interferons have been observed in response to *Pseudomonas*^10^^,^^11^ and viral infection^12^^,^^13^ in the CF lung, however the role of the respiratory epithelium in response to fungal infection is poorly understood. The aims of this study were to examine the impact of ETI CFTR modulator therapy on the clinical outcome of Aspergillus-related lung disease in people with CF and further analyse the CFTR-dependent epithelial antifungal interferome in response to Aspergillus. We provide insights into CFTR-dependent regulation of key interferon stimulated genes that drive downstream fungal infection susceptibility in CF and analyse the role of exogenous IFN administration as a potential translational therapeutic on neutrophil antifungal killing.

## Methods

### Ethics

Study participants for in vitro studies provided full informed consent and were recruited as part of two longitudinal studies; the Fungal Resistance Evolution and Acquisition in Chronic Lung Disease (FREAL) study (IRAS ID: 244685; REC ref: 19/LQO/1663), an 18-month longitudinal observation study involving patients with CF and other chronic lung diseases and Targeting Immunotherapy in Fungal Infections in Cystic Fibrosis (TRIFIC) (IRAS ID: 270828; REC ref: 20/LO/0110), a multi-centre cohort study investigating immunotherapeutic options in people with CF related fungal infection. Retrospective electronic health record data analysis was performed as part of FADE (Analysing Fungal Epidemiology in the UK) (IRAS ID: 300045; REC reference: 21/HRA/3400). For the retrospective data analysis, consent requirement was waived by approval body given clinical data and pseudoanonymised. The manuscript adheres to the 2024 Declaration of Helsinki ethical principles.

### Clinical recruitment

Clinical demographics (age, sex, CF mutation status) were recorded for adult patients (>16 years) attending a single UK CF centre before and after the introduction of highly-effective CFTR modulator therapy (Elexacaftor/Tezacaftor/Ivacaftor). Impact of CFTR modulator therapy on requirement for anti-fungal therapy, inhaled or oral corticosteroids, microbiology and *Aspergillus* serology was analysed. Study inclusion criteria was individuals with CF > 16 years receiving ETI therapy for a 12-month period and the availability of Aspergillus biomarkers in the study analysis period pre and post introduction of ETI introduction which was collected from available electronic health records with data collection between 2019 and 2022. 348 study subjects with available Aspergillus biomarker data were included in subsequent analysis. Formal sample size collection was not performed given the retrospective nature of the study. Most individuals with homozygous and heterozygous F508del were already on Tezacaftor/Ivacaftor at the time of ETI introduction so analysis reflects impact of ETI therapy substitution. Peak levels were used for analysis 12 months prior and post introduction of CFTR modulator therapy (a three-month period either side of the required date was allowed with a minimum of 9 months between pre and post values). Pairwise statistical comparison was performed by Wilcoxon matched-pairs signed rank test for serological measures with chi-squared analysis for proportion analysis.

### Fungal culture, harvest and preparation

The fungal strains used during this project were *Af* CEA10 (FGSC A1163) and Discosoma spp. red fluorescent protein (dsRed) *Af*^14^ from the Fungal Genetics Stock Centre. Fungi were grown on sterile potato dextrose agar (PDA; Oxoid, CM0041) in a T25 flask (ThermoFisher) for 4–5 days. Resting conidia were harvested in 10 ml sterile PBS (Gibco) 0.1% TWEEN (Sigma). Harvested conidia were filtered through Miracloth (Calbiochem, UK), wash and resuspended in PBS. To prepare hyphae the resting conidia were incubated at 37 °C with 5% CO_2_ for 9 h in clear RPMI, collected using PBS 0.1% TWEEN and resuspended in PBS. Hyphae were fixed in 4% paraformaldehyde (PFA) overnight, neutralised with 0.1% NH_4_Cl, washed and resuspended in PBS. Heat killed conidia were prepared by placing resting conidia in a water bath at 90 °C for 1 h.

### Epithelial cell culture and treatment of CF BEC with CFTR modulator agents

CFBE41o^−^ which is a CF human bronchial epithelial cell line (RRID:CVCL_VP35; SCC151, Merck), derived from a CF patient homozygous for the ΔF508 CFTR mutation and immortalised with the origin-of-replication defective SV40 plasmid (pSVori-) and CFBE41o^−^ 4.7 (RRID:CVCL_VP34; SCC159, Merck) is a subclone derived from the electroporation of the parental CFBE41o-cell line with an Epstein–Barr virus (EBV)-based episomal pCEPβ vector containing the 4.7 kb wild-type CFTR open reading frame (ORF) cDNA and a Hygromycin B resistance gene were grown at 37 °C with 5% CO_2_ in Minimum Essential Medium α (MEM α, Gibco) 10% (v/v) FBS. These cell lines have been previously validated with regards to CFTR function but further formal validation was not conduced as part of this study. The cell CF BECs and CF-corrected BECs were used in cultures with CF BECs further treated with a combination treatment of ETI at a 1:1:1 ratio at 0.33 ng/μl each. Treatment was carried out 24 h prior to infection by removing media, washing the cells twice with warm PBS and replenishing the media with the appropriate concentration of modulator. Epithelial cells were seeded at 5 × 10^5^ cells/ml in 12 well plates for RT-PCR and 5 × 10^4^ cells/ml in 96 well plates for LDH assays 24 h prior to infection. After 24 h, stimulation conidia or hyphae at a stated Multiplicity of Infection (MOI) or poly (I:C) at 100 μg/ml in MEM without FBS for RT- PCR and clear MEM for LDH assays. The cells were incubated for the indicated time at 37 °C with 5% CO_2_ before the supernatant and cells were harvested. Uninfected cells were treated with PBS and served as a negative control.

### RNA extraction, RT-PCR, RNA sequencing, data processing, genome alignment and normalisation

RNA was extracted from CF and CF corrected BECs (RNeasy kit, Qiagen) with an optional DNase step (RNase-free DNase Set, Qiagen) and QIAshredder columns used for homogenisation (QIAshredder, Qiagen). RNA concentration, OD260/280, and OD260/230 values were quantified using a NanoDrop 8000 UV–Vis Spectrometer (Thermofisher) and some sample quality was confirmed on a 4150 Tapestation System (Agilent). Samples with >200 ng of RNA, OD 260/280, and OD 260/230 values of >1.8, and <2.2 and RIN values >8 were sent for RNA sequencing by Novogene, Cambridge. 1 μg of isolated RNA was used for cDNA synthesis (Omniscript RT kit, Qiagen). RT-PCR was performed on a QuantStudio 3 (Applied Biosciences) using QuantiTect Probe PCR Kit (Qiagen) according to manufacturer's instructions. Results were analysed using the Thermofisher cloud. Each gene was normalised against *18S RNA* and data was converted into fold change by calculating 2- (ΔΔCt). Details of RT-PCR primers are provided in the Supplementary Materials. RNA sample quality control (QC) was completed by Novogene prior to library preparation. Preliminary QC and sample quantification, integrity and purity were carried out using Agarose Gel Electrophoresis, Bioanalyzer (Agilent 2100), Qubit Fluorometer, and Nanodrop. Novogene's Eukaryotic mRNA sequencing was carried out using the Illumina Novoseq6000 platform with paired end 150 bp, 20 million raw reads per sample. Further details are provided in the Supplementary Materials. RNA sequencing was carried out after stimulation with poly (I:C) stimulation and exposure to *Aspergillus* conidia and hyphae in CF BECs and corresponding gene corrected BECs expressing wild-type CFTR (CF-corrected BECs). Across all networks, all genes related to ribosomal function and cell growth were removed in order to focus the analysis to the immune response.

### Polymorphonuclear isolation and exogenous interferon stimulation

Blood for polymorphonuclear (PMN) cell isolation was collected in LH whole blood tubes and processed quickly after collection. 5 mls of Polymorphprep (Progen) were added to 15 ml tubes and 5 ml of whole blood was carefully layered on top. The tubes were spun at 500 g for 30 min at 20 °C with the brakes off. After the spin was complete the PMN and PBMC fractions were collected separately, washed with cold PBS, and spun at 1400 rpm for 7 min at 4 °C. Red blood cell (RBS) lysis buffer was added to the PMNs for a maximum of 10 min and the cells were washed twice with cold PBS and used straight after isolation. Demographic details for CF and healthy controls for neutrophil experiments are provided in the Supplementary Material.

### ‘Neutrophil infection and interferon treatment’

Colony Forming Unit (CFU assay): cells were plated at a final concentration of 1 × 105 cells/well in 500 μl of RPMI 1% PenStrep in a 24-well plate and infected with MOI = 0.5 live resting CEA-10 conidia. After 3 h of infection the cells were lysed using 0.05% PBS-TWEEN and the lysates moved into 1.5 ml lo-bind Eppendorf tubes. Dilutions were then carried out using DPBS to create 1:10, 1:100, and 1:1000 dilutions. Triplicates of 100 μl of neat lysate and each dilution were pipetted and spread onto Sabouraud agar on 90 mm Petri dishes. The plates were then incubated at 37 °C for 24 h. After 20 h, colonies were visible but were not overlapping. Colonies were counted manually.

Reactive oxygen species analysis: The DCFDA cellular ROS assay kit (Abcam) was used to assess ROS production in neutrophils. Freshly isolated neutrophils were pre-treated with IFNβ or IFNλ1 at appropriate concentrations for 30 min at 37 °C. Cells were stained with 10 μM DCFDA solution for a further 30 min at 37 °C in the dark. Cells were then placed in a black-walled and clear-bottomed 96-well plate at a final concentration of 5 × 10^4^ with RPMI 1% PenStrep without phenol red and infected with fixed swollen conidia at an MOI = 1. The plate was measured immediately on an Infinite F200 Fluorescence Microplate reader (Tecan) at Ex/Em = 485/535 nm for 4 h.

Neutrophil extracellular trap (NETosis) assay: 3.75 × 10^4^ dsRed Af conidia were swollen in a black-walled and clear-bottomed 96-well plate for 6 h in clear RPMI in the incubator at 37 °C. Freshly isolated neutrophils were pre-treated with IFNβ or IFNλ1 at 0.1 ng/ml, 1 ng/ml or 10 ng/ml for 30 min and 3.75 × 10^4^ were stimulated with 20 ng/ml PMA (Sigma) or placed in wells containing swollen dsRed Af conidia and incubated at 37 °C in 200 μl RPMI with 1% PenStrep for 3 h. After 3 h, the plates were gently centrifuged for 3 min, and the media was removed and stored at −80 °C. The cells and fungus were fixed with 2% PFA for 10 min at 4 °C and stained with 0.1% SytoxGreen nucleic acid stain (Thermofisher) for 20 min in the dark at room temperature. The cells were then imaged using a Cell Discoverer 7 (Zeiss) and images were analysed using the ImageJ Fiji software (https://fiji.sc/).

### Statistics

All in vitro experiments were repeated in triplicate and statistical significance was assessed with student t-test for 2-way comparison or one-way ANOVA for 3 or more experimental groups with Benjamini–Hochberg testing to correct for multiple comparisons. Heatmaps of differentially-expressed genes (DEGs) were created using a multiple comparison (one-way ANOVA) of log_2_ 2 fold changes in gene expression. Expression of top 2000 genes were cross referenced to genes set encoding Type I and Type III ISG from the Molecular Signatures Database (Human MSigDB v2022.1.Hs updated August 2022). Interferome heatmaps of DEGs were organised by hierarchical clustering based on mean gene expression and statistical significance (p adj <0.05), calculated using ANOVA and Benjamini–Hochberg tests. Volcano plots were used to illustrate DEGs with more than log_2_ 2-fold difference in expression and corrected p values <0.05 determined by Student's t-test and Benjamini-Hockberg analysis. Gene set enrichment analysis (GSEA) was carried out on Qlucore using Hallmark gene sets and the ImmuneSigDB subset of the immunologic signature gene sets from the Molecular Signatures Database. All GSEA was conducted as a comparison between two conditions and gene sets with a corrected p value (Benjamini–Hochberg test) of <0.05 were regarded significant. Cytoscape StringApp v2.0.0 analysed was used to analysis the protein network and functional enrichment on variable lists developed on Qlucore with significance thresholds set log_2_ 2-fold change or more and adjusted p value less than 0.05 (Benjamini–Hochberg test). Enhanced Graphics v1.5.5 app was used for chart visualisation of the functional enrichment terms and to display protein–protein networks. Two-way ANOVA was used for analysis of neutrophil reactive oxygen species (ROS) data and student's t-test for other assays of neutrophil function. All statistical analysis, apart from transcriptomic analysis, was carried out using GraphPad Prism version 9 and all transcriptomic statistical analysis completed using Qlucore Omics Explorer version 3.8. Details regarding further bioinformatic analysis, are available in Supplementary Materials.

### Role of funders

The funders had no role in study design, data collection and analysis, decision to publish, interpretation, or preparation of the manuscript.

## Results

### Impact of ETI CFTR modulator therapy on Aspergillus-related lung disease in individuals with CF

**Fig. 1:**
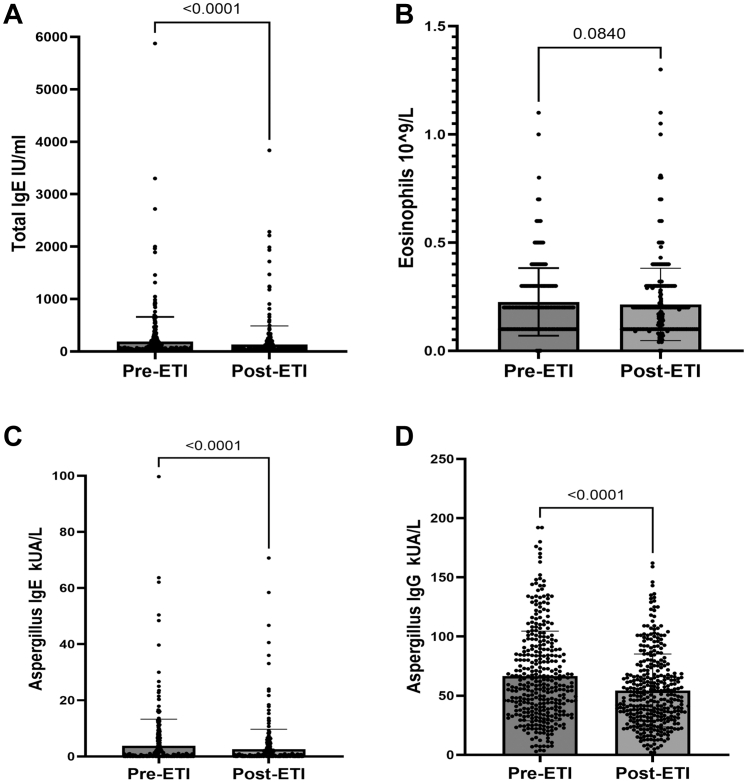
Graphs (A, B, C and D) showing a significant decrease in Aspergillus disease biomarkers (peak level) 12 months in individuals with CF (n = 384) post introduction of CFTR modulator therapy but no significant difference in eosinophil counts. Error bars represent SD. ∗p < 0.05, ∗∗p < 0.01, ∗∗∗p < 0.001.

Introduction of CFTR modulator therapy (Elexacaftor/Tezacaftor/Ivacaftor (ETI)) was associated with a significant reduction in the concentration of *A. fumigatus-*specific immunoglobin E (IgE) and G (IgG) and the proportion of people with positive Aspergillus respiratory microbiology at 12 months (Table 1 and Fig. 1) in a retrospective analysis of a large single-centre adult CF cohort (n = 348). There was also a decreased requirement for anti-fungal therapies as well as oral and inhaled corticosteroid (Table 1), up to a year after introduction of ETI modulator therapy. 30 individuals (8.6%) met the ISHAM definition of allergic bronchopulmonary aspergillosis (ABPA) based on serology at the time of ETI modulator therapy initiation, which reduced to 17 (4.9%) post ETI therapy despite the decreased requirement of antifungal and corticosteroid therapy.

**Table 1:** Cohort demographics.

Clinical demographics (n = 384)	Pre-ETI	Post-ETI	p-value
Age (years) (mean ± SD)	36.4 ± 11.5		
Female (n (%))	174 (50)		
Male (n (%))	174 (50)		
Genotype (n (%))			
*F508del/F508del*	182 (52)		
*F508del/other*	147 (42)		
*Other eligible genotypes*	17 (5)		
Antifungal treatment (n (%))	33 (10)	16 (5)	0.01
Oral corticosteroids (n (%))	52 (15)	32 (9)	0.02
Inhaled corticosteroids (n (%))	228 (66)	201 (58)	0.04
Sputum Fungal culture positive (n (%))	105 (31)	35 (11)	0.0001

The data shows a significant reduction in oral and inhaled corticosteroids, antifungal therapy and sputum fungal microbiological growth post CFTR modulatory therapy introduction.

### Analysis of the CFTR-dependent bronchial epithelial cell interferome following Aspergillus stimulation

**Fig. 2:**
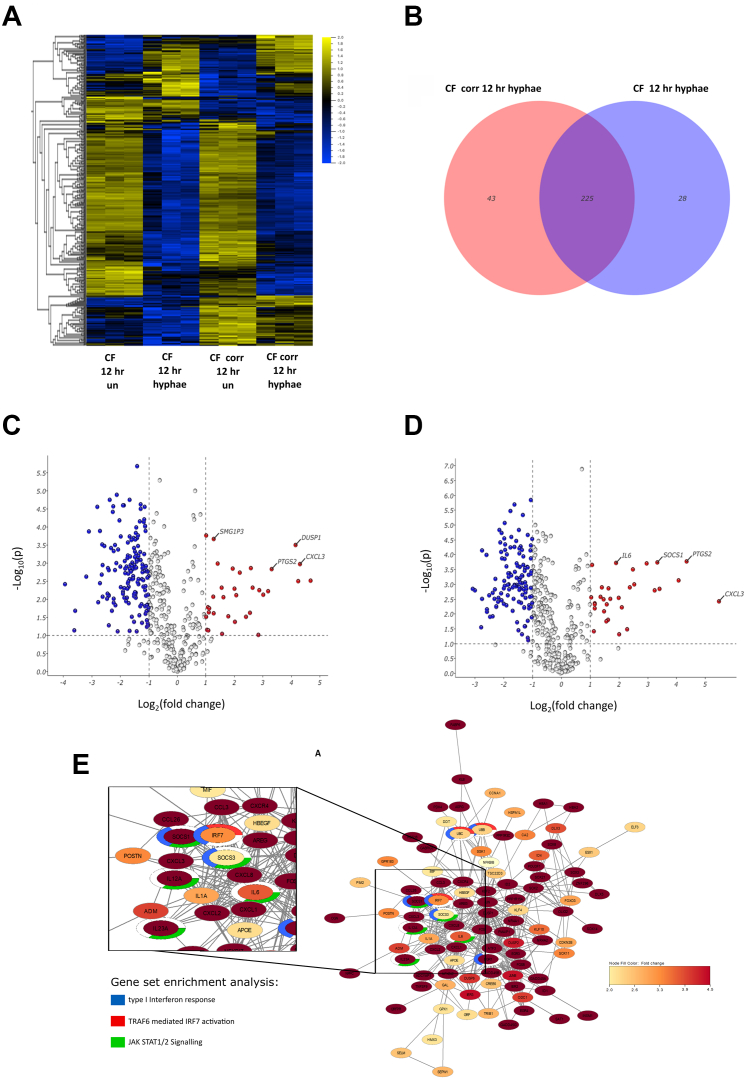
CF and CF corrected BECs were infected with *Af* hyphae (MOI = 8) for 12 h, RNA was isolated and sent for bulk RNA sequencing. (A) Heatmap was organised by hierarchical clustering based on mean gene expression and includes genes from a list of 486 ISGs. Each column represents a sample (n = 3), and each row represents a gene (padj <0.05). Significance was calculated using ANOVA and Benjamini–Hochberg test to correct p-value. (B) Venn diagram of differentially expressed ISGs compared to unstimulated control of named condition represents overlap of ISG signature in CF and CF corrected BECs. (C and D) Volcano plots represent differentially expressed ISGs for (C) CF cells and (D) CF corrected BECs infected by *Af* hyphae for 12 h, determined by student's t-test padj <0.05 and fold change >2. (E) Network visualisation of significant immunity-associated proteins (padj <0.05 and fold change >2), after 12 h of fixed hyphae infection in CF corrected BECs. The nodes indicate genes, and the colour represents fold change. Functional enrichment analysis highlights the nodes involved in the type I IFN response (blue), TRAF6 mediated IRF7 activation (red) and JAK-STAT1/2 signalling (green) (Cytoscape, stringApp). Data is from 3 experimental replicates (n = 3).

Although the effects of ETI CFTR modulator therapy on mucociliary clearance and airway infective bioburden are well established, the impact on epithelial antifungal immune responses are not well defined. To understand whether the observed clinical response seen post ETI CFTR modulator in Aspergillus-related lung disease relates in part to effects on epithelial antifungal immunity, we utilised an in vitro BEC model to analyse the CFTR-dependent interferome following Aspergillus infection. Gene expression heat maps of the top 2000 genes differentially expressed after poly (I:C) stimulation and Aspergillus conidia and hyphal infection revealed significant difference in genes related to cell death, recruitment and activation of immune cells, oxidative stress, DNA repair and epithelial cell integrity in comparison to baseline controls. We cross referenced 486 type I and III interferon-stimulated genes (ISGs) using multiple genes set from the Molecular Signatures Database. Transcriptomic characterisation of ISGs in CF and CF-corrected BECs following Aspergillus hyphal stimulation showed 361 differentially expressed genes (padj = 0.05 (ANOVA)) in infected BECs compared to uninfected cells indicating significant CFTR-dependent regulation of interferon gene expression during fungal infection (Fig. 2A and Suppl Figure S1A). There were 43 differentially expressed ISGs unique to CF-corrected BECs (Fig. 2B) in comparison to CF BECs. Using a more stringent filtering criteria (log 2 [fold chang] >2 and p-adjusted value <0.05 (Anova)) we show clear differences in ISG responses to fungal stimulus between CF and CF-corrected BECs (Fig. 2C–D, Table 2). The top five ISGs upregulated in CF BECs included genes encoding chemokines for neutrophil recruitment, activation and resistance to apoptosis (*CXCL2*, *CXCL3*, *CXCL8*),^15^ *Prostaglandin-endoperoxidase synthase 2* (*PTGS2*), which is linked to pro-inflammatory immune responses in CF^16^ and *Dual Specificity Phosphatase 1* (*DUSP1)*, a target of Vitamin D mediated down-regulation of IL-8.^17^ Biological activity of comparable ISGs in CF-corrected BEC were more wide-ranging including genes encoding for *Colony Stimulating Factor 2* (*CSF2*), critical for up-regulation of neutrophil-dependant *Aspergillus* killing,^18^ neutrophilic inflammation (*CXCL2*), *APOBEC3G* a retroviral restriction factor,^19^ *IL-7R* (cytokine involved in T cell development and maturation and proposed to have a complex role in fungal asthma),^20^^,^^21^ and *Hydroxycarboxylic acid receptor 3* (*HCAR3*), an anti-inflammatory protein active in macrophages and neutrophils.^22^ Similar gene families encoding metabolic and anti-inflammatory proteins were down-regulated in CF-corrected and CF BECs although changes were more pronounced in the latter (Table 2). Gene set enrichment analysis (GSEA) of the CFTR-dependant interferome following hyphal stimulation showed significant enrichment for the interferon-related set ‘Hecker IFNB1 Targets’ (NES = 1.54, padj = 0.01) and the ‘Reactome TRAF6 Mediated IRF7 Activation’ (NES = 1.76, padj = 0.05) without any significant changes in interferon signalling pathway gene sets in the CF BECs (Fig. 2E and Suppl Figure S1A). Network visualisation of genes encoding immunity-associated proteins after 12 h of fixed hyphae infection showed significantly greater upregulation of proteins associated with TRAF-6 mediated *IRF7* activation, JAK-STAT1/2 signalling and Type I IFN immune responses in CF-corrected BECs such as *SOCS1*, *SOCS3*, *EGR1* and *IL12A* and *IL23A* (Fig. 2E). In contrast only Type I IFN immune responses were observed in CF BECs (Suppl Figure S1B). Transcriptomic characterisation of ISGs in CF and CF-corrected BECs following Aspergillus heat killed conidia stimulation showed similar though less marked changes in interferon responses compared to fixed hyphae (Suppl Figure S2A–D, Suppl Table S1). Network visualisation of genes encoding immunity-associated proteins showed enrichment for the interferon-related set ‘Hecker IFNB1 Targets’ (padj = 0.07; NES = 1.73) and ‘Hallmark Inflammatory Response’ (padj = 0.01; NES = 1.67) in CF corrected BECs and no change in CF BECs.

**Table 2:** The top five upregulated and downregulated ISGs in CF and CF corrected BECs after fixed hyphae infection for 12 h.

Overlapping genes are highlighted in bold, upregulated genes are in red cells and downregulated in blue (padj <0.05; fold change >2).

To determine if the defect in the type I and III IFN response following fungal stimulation observed in transcriptomic analysis was due to a downregulation in the expression of IFNβ and IFNλ1 in CF cells, as observed in Pseudomonal bacterial and viral infections, RT-PCR of infected CF and CF corrected cells was carried out to assess the expression of these genes directly. *IFNβ* and *IFNλ1* expression was significantly increased in CF corrected BECs compared to CF BECs, however *IL-8* expression did not differ (Suppl Figure S3). Similar defects within gene and transcriptomic analysis were seen following poly:IC stimulation with further details and results provided in the Supplementary Material (Suppl Figure S4 and Suppl Table S2).

### CFTR modulator therapy partially restores the type I and III interferon response in CF BECs following Aspergillus stimulation

**Fig. 3:**
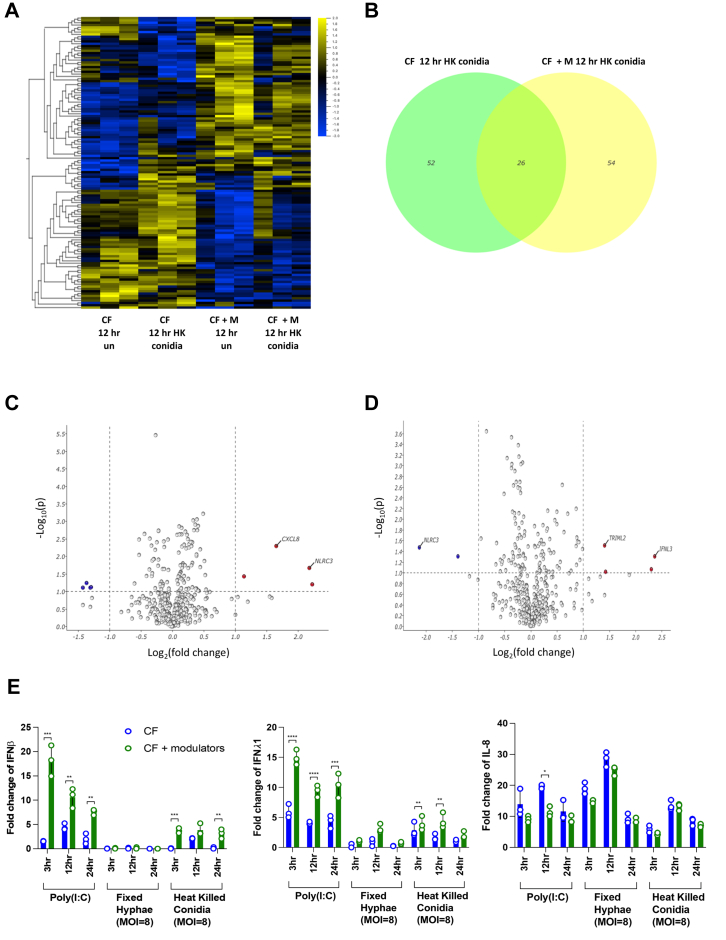
CF BECs were treated with combination modulator treatment consisting of 1 ng/ml of tezacaftor/ivacaftor/elexacaftor (1:1:1 ratio) for 24 h prior to, and again alongside Af heat killed conidia infection (MOI = 8) for 12 h. (A) Heatmap was organised by hierarchical clustering based on mean gene expression and only includes genes from a curated list of 486 ISGs. Each column represents a sample (n = 3), and each row represents a gene (padj <0.05). Significance was calculated using ANOVA and Benjamini–Hochberg test to correct p-value. (B) Venn diagram of differentially expressed ISGs compared to uninfected control of named condition representing overlap of ISG signature in CF BECs with and without modulator treatment. (C and D) Volcano plots representing differentially expressed genes from the same list of 486 ISGs for Af infected CF cells without modulator treatment (C) and with modulator treatment (D) and, determined by student's t-test padj <0.05 and fold change >2. (E) IFNβ, IFNλ1 and IL-8 RT-PCR expression by CF BECs after stimulation with poly (I:C) (10 μg/ml), fixed Af hyphae (MOI = 8) and heat killed Af conidia (MOI = 8) for 3, 12 and 24 h with and without combination CFTR modulator therapy (pre-treatment as described above). Data is from 3 experimental replicates (n = 3).

We further analysed how ETI CFTR modulator therapy impacts the CF epithelial antifungal interferome response. 216 ISGs were differentially expressed in CF BECs with and without ETI CFTR modulator treatment following stimulation with heat-killed Aspergillus conidia as calculated by ANOVA (padj = 0.05) (Fig. 3A). There were 52 differentially expressed ISGs in the CF BECs infected and 54 differentially expressed ISGs in CF BECs treated with ETI CFTR modulator therapy compared to uninfected controls with 26 shared ISGs (Fig. 3B). Differentially expressed ISGs are graphically represented in volcano plots (Fig. 3C and D) and detailed in Table 2. *Interferon Lambda 3* (*IFNL3*) was significantly increased and *NLRC3* was significantly reduced in CFTR modulator treated CF BECs (Fig. 3C and D and Table 3). In contrast, *IFNL3* was downregulated and *NLRC3* was upregulated in CF BECs without modulator therapy. GSEA showed no enhancement of ISG pathways in CF BECs with and without ETI modulator therapy. There were no ISGs present in the significantly upregulated gene networks after Aspergillus stimulation in CF BECs. After ETI modulator treatment, 3 out of the 5 upregulated genes in the protein–protein network were type I ISGs (Suppl Figure S5). RT-PCR validation revealed significantly higher *IFNβ* and *IFNλ1* expression (5-fold increase) in CF BECs with ETI CFTR modulator treatment following heat-killed Aspergillus conidia stimulation (Fig. 3E). Administration of ETI modulator therapy in vitro did not result in any change in expression of *IFNβ* and *IFNλ1* following Aspergillus hyphal infection in CF BECs. Similar upregulation of ISGs was seen with ETI modulatory therapy within transcriptomic analysis following poly:IC stimulation (Suppl Table S3).

**Table 3:** All differentially expressed ISGs in CF BECs after heat killed conidia infection for 12 h with and without CFTR modulator treatment.

Overlapping genes are highlighted in bold, upregulated genes are in red cells and downregulated in blue (padj <0.05; fold change >2).

### Exogenous IFN-λ1 administration rescues CF neutrophil antifungal killing without exacerbating immunopathology

**Fig. 4:**
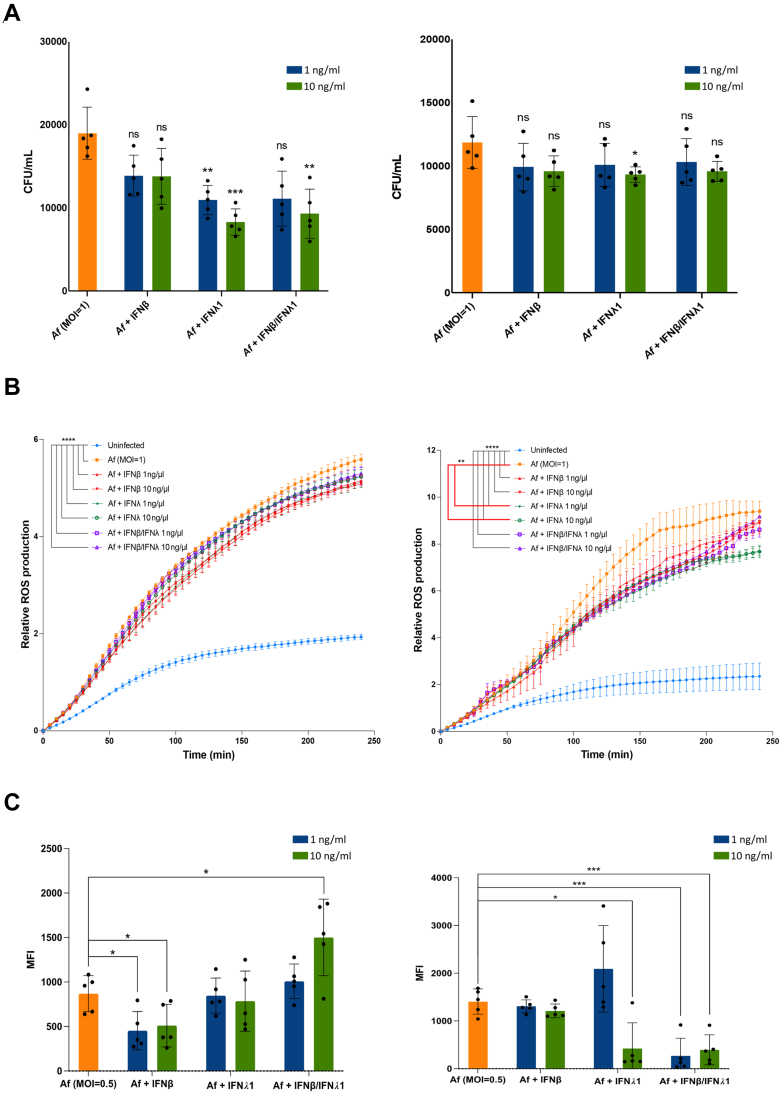
The effect of exogenous IFNβ and IFNλ1 on CF and healthy neutrophil fungal killing. Neutrophils were isolated from five CF and healthy donors and then treated with either IFNβ, IFNλ1 or both together at a concentration of 1 ng/ml and 10 ng/ml both in the presence and absence of Af infection (conidia) at an MOI = 0.5. A) CFUs were obtained from lysates of neutrophils after 3 h of infection in the presence of exogenous IFN treatments. B) ROS production was quantified using a Tecan plate reader over 4 h (240 min) in the absence of fungal infection and the addition of live swollen Af conidia at an MOI = 1. (C) NETosis was measured after 3 h of exogenous IFNβ, IFNλ1 or both together with and without live swollen Af infection (MOI = 0.5) by staining with SYTOXGreen DNA stain and imaging using a Zeiss CD7 fluorescent microscope. MFI of the SYTOXGreen was calculated and background of cells alone was removed from images without fungus and data was presented as a measurement of extracellular NETs. Where Af infection was present, a second normalisation step was carried out to remove the fluorescence of the fungus, therefore MFI measurements of images with Af alone were also subtracted and data was presented as a measurement of extracellular NETs. Statistical significance was tested with repeated measures ANOVA for ROS data and one-way ANOVA with Benjamini–Hochberg testing to correct for multiple comparisons for the rest of the data. Data is from 5 biological replicates (n = 5). Error bars represent SD. ∗p < 0.05, ∗∗p < 0.01, ∗∗∗p < 0.001, ∗∗∗∗p < 0.0001.

Although our study indicates fungal-related lung disease outcomes have improved in CF following ETI CFTR modulator therapy introduction, our prior results suggest only a partial correction of antifungal immune responses with ongoing concern additionally related to individuals not suitable or tolerant of ETI modulator therapy. Given our data above indicating a diminished ISG expression in CF epithelial cells following fungal infection, we analysed the effects of exogenous IFNβ and IFNλ1 on neutrophil responses isolated from people with CF following infection with *A. fumigatus* live conidia. Administration of IFNλ1 at 10 ng/μl led to a significant increase in fungal killing in CF neutrophils, with administration at 1 ng/μl and 10 ng/μl increasing neutrophil fungal killing from healthy controls (Fig. 4A). Administration of IFNβ alone (1 and 10 ng/μl) had no impact on fungal killing. Given the importance of neutrophil-derived reactive oxygen species (ROS) production and neutrophilic extracellular trap (NET) formation in exacerbating immunopathology in CF, we analysed effects of exogenous type I and III interferons on these pathways. In CF and healthy neutrophils, Aspergillus conidial infection led to a significant increase in ROS production compared to uninfected neutrophils. Administration of IFNλ1 (1 ng/μl and 10 ng/μl) significantly reduced ROS production in Aspergillus infected CF neutrophils (Fig. 4B). No significant differences in ROS production were seen in IFNβ treated CF neutrophils. Additionally, pre-treatment with IFNλ-1 10 ng/ml and the combination of IFNλ-1/IFNβ (1 ng/ml and 10 ng/ml) significant reduced NETosis in Aspergillus infected CF neutrophils, with IFNβ administration (1ng/10 ng/ml) not resulting in any significant change in NETosis in Aspergillus infected CF neutrophils compared to infected controls. This was contrary to healthy neutrophils where a reduction in NETosis was noted with IFNβ pre-treatment.

## Discussion

In this study we show that ETI CFTR modulator therapy improves fungal infection-related outcomes in people with CF with a reduction in biomarkers of *A. fumigatus* infection and a reduction in anti-fungal and corticosteroid therapy requirement. We demonstrate a potential immune mechanism for this clinical improvement through CFTR-dependent downregulation of the epithelial interferome in response to *A. fumigatus* stimulation with partial correction following ETI modulator therapy. Exogenous administration of IFNλ1 in vitro augmented the capacity of neutrophils isolated from people with CF to eliminate *A. fumigatus* spores while also reducing excessive potentially harmful pro-inflammatory immune responses, providing a rational basis for the development of future targeted therapeutic approaches in CF-related fungal disease.

Triple combination CFTR modulator therapy (ETI) has led to marked improvement in clinical outcomes in people with CF. In comparison to bacterial infection, however there is relatively little data on the CFTR-dependent antifungal immune response and fungal infection related clinical outcomes following CFTR modulator therapy introduction.^23^^,^^24^ Our data confirms a recent report analysing Aspergillus airway burden through molecular detection methods,^25^ and is similar to that seen in people with CF on ivacaftor therapy.^26^ Analysis based on sputum cultures is limited by reduced sputum production post ETI therapy, however our findings of improved serology biomarkers and therapeutic requirement for antifungal and corticosteroids is supportive of improved Aspergillus disease-related outcomes. The majority of individuals within the study at the time of ETI introduction with homozygous or heterozygous F508del were already on Tezacaftor/Ivacaftor so our findings likely underestimate the complete effects of CFTR modulator therapy. Improvement in total and Aspergillus IgE levels are however in contrast to the lack of effect on eosinophils. Whether this relates to reduced antigen load secondary to improved mucociliary clearance and anti-fungal immune responses, without effects on epithelial epigenetic drivers of eosinophilic inflammation is not clear and requires further investigation. The impact of modulator therapy on the epithelial immune response and in particular the interferon response is less well understood in fungal infection compared with bacterial (Pseudomonal) and viral infection in CF.^27^ Our results confirm within a host-pathogen setting the relevance of previous findings of partial reversal of Type I/III IFN defects in epithelial cells following triple CFTR modulator therapy in children with CF.^28^

Respiratory epithelial cells play an important role in the initial host immune response to *A. fumigatus*. Although less well studied than in alveolar macrophages, CFTR-dependent epithelial pathways facilitate the clearance of Aspergillus spores, maintain epithelial cell viability and inhibit exaggerated immune responses to fungal hyphae.^29^ Aspergillus conidia can induce interferon-β signalling in human bronchial epithelial cell cultures in a TLR3/Receptor-Interacting Protein (RIP)-1/TANK-binding kinase signalling pathway.^30^ Murine CF studies have previously shown that respiratory epithelial cells secrete CXCL chemokines to recruit neutrophils and GM-CSF growth factor to enhance Aspergillus conidial killing in an IL-1 and type III interferon manner.^9^^,^^31^ TLR-4/TRIF induced type I interferon secretion is also described in CF respiratory epithelial cells,^32^ with pulmonary type I IFN responses following live Aspergillus challenge primarily driven by TLR3/TRIF dependent signalling whereas activation of the MDA5/MAVS pathway promote a type III IFN response.^33^ Similar pathways operate in a human BEC model and our data show the relevance of epithelial cell culture and murine models for the mechanistic understanding of human disease. Our study showed broadly similar transcriptional IFN responses between fungal hyphae and heat-killed conidia however at present there is little understanding of how fungal germination shapes the epithelial antifungal interferon response and further research is required to better delineate dynamics. This study provides evidence for the contribution of the interferon signalling pathway in host defence to fungal infection and highlights a potential novel role of CFTR regulation of TRAF6-mediated IRF7 activation, which in turn is a critical regulator of type I IFNs [33] and *NLRC3*, a negative regulator of the cGAS-STING pathway and NLRP3 inflammasome to modulate inflammatory signalling pathways.^34^ Previous studies have highlighted the importance of the cGAS-STING pathway in antifungal responses within murine and human bronchial epithelial cell models. Further studies are required to elucidate a more detailed mechanistic understanding of impaired type I/III interferon immunity in CF.

Despite the improved outcomes in people in CF following introduction of ETI modulator therapy, challenges remain with regards to suitability, tolerability and as demonstrated in our study only partial restoration of anti-infective immune responses. In our study, we highlight the potential translational relevance of the type I and III IFN defect with exogenous IFNλ1 treatment of CF neutrophils resulting in increased *A. fumigatus* killing without increasing inflammatory ROS or NET production. Our results concerning ROS production differ from those previously reported in murine models of Aspergillus infection, explainable in that human neutrophils use different mechanisms to kill *Aspergillus* conidia and hyphae; generation of ROS to eliminate hyphae and a PI3K-dependent non-oxidate pathway involving lactoferrin sequestration of iron to kill conidia.^35^^,^^36^ Further studies of mechanism of action of exogeneous IFNλ1 on neutrophil anti-fungal effector function should assess if this cytokine influences non-oxidate neutrophil-mediated Aspergillus conidial killing. Further analysis of effects of exogenous IFN on neutrophilic inflammation including inflammatory cytokines is also required to determine whether the reduction in ROS and NET production relates to improved fungal killing only, or secondary to further immune modulatory effects on ROS-induced NETosis as previously postulated.^37^ Our data therefore suggests a plausible immunotherapeutic option to improve outcomes from fungal infection in people with CF without promoting harmful pro-inflammatory responses, although in vitro results will need to be confirmed in larger studies. Pegylated IFNλ has improved clinical outcome in SARS-CoV-2 infection and could be considered to boost wider defective anti-viral and antifungal interferon-dependent immunity in CF.^38^

Our study has a number of limitations including reliance on a CF in-vitro epithelial model and a relatively short term (12 months) of follow-up of clinical data from a single CF cohort with the presence of a number of confounding factors limiting an ability to infer causality. The effects of CFTR modulator therapy are additionally wide-ranging and although impact on infection burden will be undoubtedly related to a number of host factors, understanding effects on the host immune response is critical. Further studies to confirm findings derived from in vitro transcriptomic analysis and inclusion of proteomic data alongside wider international prospective registry studies focussed on fungal outcomes in CF post modulator therapy are important areas for future work.^39^

In summary, in our study we show that introduction of ETI CFTR modulator therapy is associated with improved *A. fumigatus* infection-related clinical biomarkers in people with CF potentially related in part to a restoration of an anti-fungal type I and III interferon host defence. Our findings provide fundamental functional insight into possible mechanisms driving fungal susceptibility in people with CF and provide a rational basis for the development of future targeted therapeutic approaches to improve outcomes of fungal infection in CF.

## Contributors

SL, DAJ, AS and PK conceived the study design. AS, IF, NS and SL enabled and performed sample acquisition and SL performed experiments and bioinformatic analysis. MC and BH performed the retrospective data analysis and verification. All authors contributed to manuscript drafting and revision and have read and approved the final version of the manuscript.

## Data sharing statement

De-identified participant data collected as part of the study including immune parameter, and transcriptomic analysis will be made available on request.

## Declaration of interests

DAJ has share options in Pulmocide Limited. AS has received research grant funding from Pfizer, Gilead sciences and Astra-Zeneca, advisory board/consultancy/speaker fees from Astra-Zeneca, Mundipharma, Argenyx, Kymera Therapeutics, Gilead sciences and Insmed. NJS has received research grant funding from the CF trust, Vertex pharmaceuticals and Royal Brompton-Kings Health Partners transformation fund and honoraria for advisory boards from Vertex pharmaceuticals, Gilead, Boerhinger-Ingelheim, Chiesi and Menarini. He has also received honoraria for educational activities and travel grants from Vertex, Pari, Chiesi, Medison, Gilead. He has leadership positions within the ECFS clinical trials network and UK CF registry. IF has received speaker fees for educational activities from Vertex Pharmaceuticals. D.A-J. also holds funding from; Wellcome Trust (no. 219551/Z/19/Z) and Medical Research Council (MRC) (grant no. MR/V037315/1). AS holds funding from MRC (grant no: MR/T005572/1 and grant no: MR/Y008863/1) and is supported by the MRC centre grant (MR/X020258/1). IF holds funding from Kings Health Partners Clinical and Academic Innovation Project, the Royal Brompton Hospital Charity and from the UK Cystic Fibrosis Trust to her institution (MATRIARCH_CF, SRC 027). PK holds funding from MRC grant (MR/W020653/1). SL, MC, BH and PK have no conflicts of interest to disclose.

## Appendix B Supplementary data

Supplementary Material

ICLAC_cell line_completed
