# Supplementary material for: CFTR modulators partially restore the epithelial interferome in Aspergillus infection to improve clinical outcome
Source: eBioMedicine. 2026 Jan 31;124:106131. doi: 10.1016/j.ebiom.2026.106131 (PMC12878693; doi:10.1016/j.ebiom.2026.106131)
Supplement: Supplementary Material [file mmc1.docx]

**Supplementary Materials**

**Primer and probe sequences used in qPCR:**

| Target gene | Sequence **(5’ – 3’)** |
| --- | --- |
|  |  |
| *IFNβ* | Forward: CGCCGCATTGACCATCTA  Reverse: TTAGCCAGGAGGTTCTCAACAATAGTCTCA  Probe: FAM-TCAGACAAGATTCATCTAGCACTGGCTGGATAMRA |
| *IFNλ1* | Forward: **GGACGCCTTGGAAGAGTCACT**  Reverse: **AGAAGCCTCAGGTCCCAATTC**  Probe: **FAM-AGTTGCAGCTCTCCTGTCTTCCCCG-TAMRA** |
| *IL-8* | Forward: **CTGGCCGTGGCTCTCTTG**  Reverse: **CCTTGGCAAAACTGCACCTT**  Probe: **FAM-CAGCCTTCCTGATTTCTGCAGCTCTGTGTTAMRA** |
| *18S RNA* | Forward: **CGCCGCTAGAGGTGAAATTCT**  Reverse: **CATTCTTGGCAAATGCTTTCG**  Probe: **FAM-ACCGGCGCAAGACGGACCAGA-TAMRA** |

**RNA Sequencing methods and analysis**

FastQC was used to assess the quality of the sequencing reads after they were returned from Novogene. HISAT2 v2.2.1 was used for alignment. The human reference genome used for alignment was Homo_sapiens.GRCh38.dna_sm.toplevel.fa.qz (2022-01-26) from Ensembl. Alignment scores exceeded 85% for all samples. After alignment using HISAT2, the output of each paired end read was in a combined “.SAM” file. SAMtools v1.16.1 was used to convert file types and the count matrix was made using the Subread v2.0.3 package feature, named “featureCounts’’. The count matrix was normalised (FPKM) and analysed using Qlucore Omics Explorer v3.8 (Qlucore, Lund, Sweden).

The following genes set, the Molecular Signatures Database (Human MSigDB v2022.1.Hs updated August 2022) called “Blanco Melo Beta Interferon Treated Bronchial Epithelial Cells” and “GOBP Response to Type III Interferon” (Human_NCBI_Gene_ID; GOC:add, ISBN:0126896631, PMID:15546383, PMID:16734557) were used to cross reference differential gene expression

**Neutrophil infection and interferon treatment**

Demographic details for CF and healthy controls for neutrophil experiments are provided below.

|  | Age | Gender  M / F | CFTR mutation | FEV1 (% predicted) | Infective pathogens |
| --- | --- | --- | --- | --- | --- |
| CF | 46 | M | F508del/  3011_3019del |  | *Aspergillus fumigatus* |
|  | 43 | F | p.Arg74Gln/  p.Arg297Gln |  | *Pseudomonas aeruginosa* |
|  | 20 | F | F508del/  F508del |  | *Staphylococcus aureus* |
|  | 32 | F | F508del/  F508del |  | *n/a* |
|  | 27 | F | F508del/  F508del |  | *Aspergillus fumigatus* |
|  | Mean (sd) = 33 (9.7) | Female (%) = 80 |  | | |
| Healthy control | 42 | M |  | | |
|  | 25 | F |  |  |  |
|  | 58 | M |  |  |  |
|  | 23 | F |  |  |  |
|  | 27 | M |  |  |  |
|  | Mean (sd) = 35 (13.3) | Female (%) = 40 |  |  |  |

**RESULTS**

**Supplementary Table 1:** Differentially expressed ISGs in CF and CF corrected BECs after stimulation with *Aspergillus* heat-killed conidia for 12 hours (data represents 3 experimental replicates). Overlapping genes are highlighted in yellow, upregulated genes are in red cells and downregulated in blue (padj<0.05; fold change >2).

| **CF corrected BECs** | | | **CF BECs** | | |
| --- | --- | --- | --- | --- | --- |
| **Gene symbol** | **P(adj)** | **Fold Change** | **Gene Symbol** | **P(adj)** | **Fold Change** |
| *CXCL8* | 0.002 | 5.43 | *FGF5* | 0.043 | 4.67 |
| *SCG5* | 0.003 | 3.81 | *NLRC3* | 0.021 | 4.51 |
| *IL24* | 0.007 | 3.54 | *CXCL8* | 0.005 | 3.13 |
| *PTGS2* | 0.025 | 2.39 | *SNORD17* | 0.037 | 2.20 |
| *ANGPTL4* | 0.049 | 2.31 | *IFNL3* | 0.039 | -2.69 |
| *CSF2* | 0.035 | 2.26 | *IDO1* | 0.047 | -2.58 |
| *MMP3* | 0.048 | 2.23 | *PLD5* | 0.048 | -2.47 |
| *KRT34* | 0.003 | 2.06 | *CADPS2* | 0.046 | -2.46 |
| *NCALD* | 0.008 | -4.41 |  |  |  |
| *TNFSF10* | 0.004 | -3.89 |  |  |  |
| *SYT12* | 0.012 | -2.01 |  |  |  |

**Supplementary Table 2:** Differentially expressed ISGs in CF and CF corrected BECs after stimulation with poly(I:C) for 24 hours (data represents 3 experimental replicates). Overlapping genes are highlighted in yellow, upregulated genes are in red cells and downregulated in blue (padj<0.05; fold change >2).

| **CF corrected BECs** | | | **CF BECs** | | |
| --- | --- | --- | --- | --- | --- |
| **Gene Symbol** | **P(adj)** | **Fold Change** | **Gene Symbol** | **P(adj)** | **Fold Change** |
| *CXCL3* | 0.004 | 5.59 | *GBP5* | 0.011 | 8.05 |
| *APOBEC3G* | 0.028 | 5.25 | *IL7R* | 0.009 | 6.52 |
| *IL7R* | 0.006 | 4.50 | *ESM1* | 0.016 | 6.06 |
| *CSF2* | 0.008 | 4.49 | *IL1A* | 0.014 | 5.55 |
| *HCAR3* | 0.015 | 4.47 | *SHC4* | 0.011 | 4.56 |
| *SAMD9L* | 0.020 | 4.27 | *IL24* | 0.041 | 4.20 |
| *PMAIP1* | 0.006 | 4.24 | *ANGPTL4* | 0.011 | 4.18 |
| *ISG15* | 0.007 | 3.98 | *IFIT3* | 0.014 | 3.85 |
| *IFIT3* | 0.005 | 3.95 | *PMAIP1* | 0.007 | 3.68 |
| *LMO2* | 0.012 | 3.85 | *ISG15* | 0.007 | 3.67 |
| *VEGFC* | 0.004 | 3.77 | *ACE2* | 0.038 | 3.27 |
| *CXCL8* | 0.014 | 3.69 | *SAMD9L* | 0.010 | 3.11 |
| *ESM1* | 0.013 | 3.33 | *TNFAIP3* | 0.048 | 3.10 |
| *SAMD9* | 0.014 | 3.19 | *CSF2* | 0.058 | 2.94 |
| *GBP5* | 0.010 | 3.07 | *HCAR3* | 0.011 | 2.92 |
| *TNFAIP3* | 0.009 | 3.02 | *HSD11B1* | 0.010 | 2.89 |
| *IL23A* | 0.006 | 3.00 | *IFIT1* | 0.044 | 2.84 |
| *DHX58* | 0.009 | 2.90 | *VEGFC* | 0.010 | 2.83 |
| *G0S2* | 0.028 | 2.84 | *BIRC3* | 0.007 | 2.81 |
| *IL6* | 0.008 | 2.79 | *ANTXR2* | 0.011 | 2.76 |
| *IFIH1* | 0.008 | 2.79 | *BATF2* | 0.013 | 2.65 |
| *PLAUR* | 0.007 | 2.67 | *G0S2* | 0.010 | 2.61 |
| *IFIT1* | 0.012 | 2.60 | *SAMD9* | 0.011 | 2.57 |
| *PLCG2* | 0.014 | 2.43 | *IFIH1* | 0.032 | 2.52 |
| *IFIT2* | 0.008 | 2.40 | *APOBEC3G* | 0.042 | 2.49 |
| *FYB1* | 0.015 | 2.35 | *CXCL8* | 0.011 | 2.46 |
| *IL24* | 0.062 | 2.31 | *PLCG2* | 0.040 | 2.42 |
| *ANTXR2* | 0.028 | 2.23 | *DHX58* | 0.042 | 2.41 |
| *BIRC3* | 0.004 | 2.23 | *IFIT2* | 0.008 | 2.38 |
| *TRPV3* | 0.045 | 2.09 | *DDX60L* | 0.018 | 2.36 |
| *CTSS* | 0.014 | 2.07 | *OAS2* | 0.010 | 2.31 |
| *USP18* | 0.048 | 2.06 | *IL23A* | 0.042 | 2.28 |
| *NFE2L3* | 0.007 | 2.05 | *PDZD2* | 0.018 | 2.25 |
| *IFNL1* | 0.012 | 2.05 | *PLAUR* | 0.014 | 2.24 |
| *NAV3* | 0.017 | 2.04 | *IL6* | 0.049 | 2.19 |
| *DAPP1* | 0.004 | 2.03 | *CTSS* | 0.050 | 2.16 |
| *RELB* | 0.008 | 2.01 | *NMNAT2* | 0.047 | 2.10 |
| *RTP4* | 0.062 | 2.01 | *FERMT1* | 0.017 | 2.09 |
| *ACE2* | 0.050 | 2.00 | *PDCD1LG2* | 0.031 | 2.00 |
| *PTPN22* | 0.018 | -3.98 | *PLD5* | 0.018 | -6.84 |
| *EDIL3* | 0.016 | -2.29 | *APOD* | 0.016 | -3.10 |
| *PDK4* | 0.014 | -2.28 | *SERPINB9* | 0.014 | -2.34 |
| *IGFBP3* | 0.045 | -2.22 | *SYT12* | 0.045 | -2.33 |
| *IRF8* | 0.015 | -2.17 | *GALM* | 0.015 | -2.27 |
|  |  |  | *IRF8* | 0.011 | -2.17 |
|  |  |  | *IGFBP3* | 0.041 | -2.11 |
|  |  |  | *PDK4* | 0.047 | -2.08 |
|  |  |  | *B4GALNT2* | 0.012 | -2.08 |

**Supplementary Table 3:** All differentially expressed ISGs in poly(I:C) stimulated CF cells with and without CFTR modulator treatment (data represents 3 experimental replicates). Overlapping genes are highlighted in yellow, upregulated genes are in red cells and downregulated in blue (padj<0.05; fold change >2).

| **CF BECs** | | | **CF BECs + Modulators** | | |
| --- | --- | --- | --- | --- | --- |
| **Gene Symbol** | **P(adj)** | **Fold Change** | **Gene Symbol** | **P(adj)** | **Fold Change** |
| *FGF5* | 0.025 | 4.75 | *PAMR1* | 0.001 | 10.48 |
| *GBP5* | 0.011 | 3.65 | *GBP5* | 0.012 | 3.95 |
| *HCAR3* | 0.014 | 3.48 | *SAMD9L* | 0.018 | 3.70 |
| *IL7R* | 0.005 | 3.15 | *HCAR3* | 0.033 | 3.69 |
| *IFIT3* | 0.006 | 2.60 | *IFIT3* | 0.001 | 3.37 |
| *SAMD9L* | 0.042 | 2.39 | *LMO2* | 0.047 | 3.08 |
| *SERPINB9* | 0.026 | 2.26 | *IFIT1* | 0.009 | 3.04 |
| *DHX58* | 0.014 | 2.17 | *IL7R* | 0.000 | 3.04 |
| *C1S* | 0.014 | 2.16 | *ISG15* | 0.000 | 3.00 |
| *PMAIP1* | 0.006 | 2.09 | *IFNB1* | 0.035 | 2.75 |
| *IFIH1* | 0.006 | 2.05 | *PLCG2* | 0.010 | 2.72 |
| *IFIT1* | 0.045 | 2.02 | *DHX58* | 0.003 | 2.54 |
| *PLD5* | 0.038 | -3.27 | *TRANK1* | 0.031 | 2.51 |
| *IDO1* | 0.019 | -2.37 | *IFIH1* | 0.003 | 2.48 |
|  |  |  | *SAMD9* | 0.021 | 2.42 |
|  |  |  | *APOBEC3F* | 0.009 | 2.26 |
|  |  |  | *ZNFX1* | 0.003 | 2.26 |
|  |  |  | *ACE2* | 0.002 | 2.22 |
|  |  |  | *IFIT2* | 0.005 | 2.12 |
|  |  |  | *APOBEC3A* | 0.042 | 2.10 |
|  |  |  | *NMNAT2* | 0.004 | 2.09 |
|  |  |  | *PLEKHA4* | 0.042 | 2.05 |
|  |  |  | *IRF1-AS1* | 0.003 | 2.05 |
|  |  |  | *IL16* | 0.019 | 2.02 |
|  |  |  | *PMAIP1* | 0.000 | 2.01 |
|  |  |  | *SHC4* | 0.038 | -3.27 |
|  |  |  | *PLD5* | 0.038 | -2.81 |
|  |  |  | *ADAMTS6* | 0.003 | -2.39 |
|  |  |  | *IL24* | 0.035 | -2.37 |
|  |  |  | *RPLP0P2* | 0.030 | -2.10 |

**Supplementary Figure 1**: A) Representative visualisation of GSEA of CF and CF corrected BECs infected with Af fixed hyphae for 12 hours (data represents 3 experimental replicates). CF corrected BECs infected with Af hyphae (MOI=8) for 12 hours showed enrichment of ‘Hecker IFNB1 Targets’ gene set (padj=0.01). CF cells did not show enrichment of this gene set (padj=0.7). Significance calculated using Student’s T-test. Gene set consists of 96 genes. Network visualisation of significant immunity-associated proteins (padj<0.05 and fold change >2), after 12 hours of fixed hyphae infection in CF BECs. The nodes indicate genes and the colour represents fold change. Functional enrichment analysis highlights a comparative reduction in nodes involved in the type I IFN response (blue), TRAF6 mediated IRF7 ac(va(on (red) and JAK-STAT1/2 signalling (green) compared to CF-corrected BECs(Cytoscape, stringApp).

**
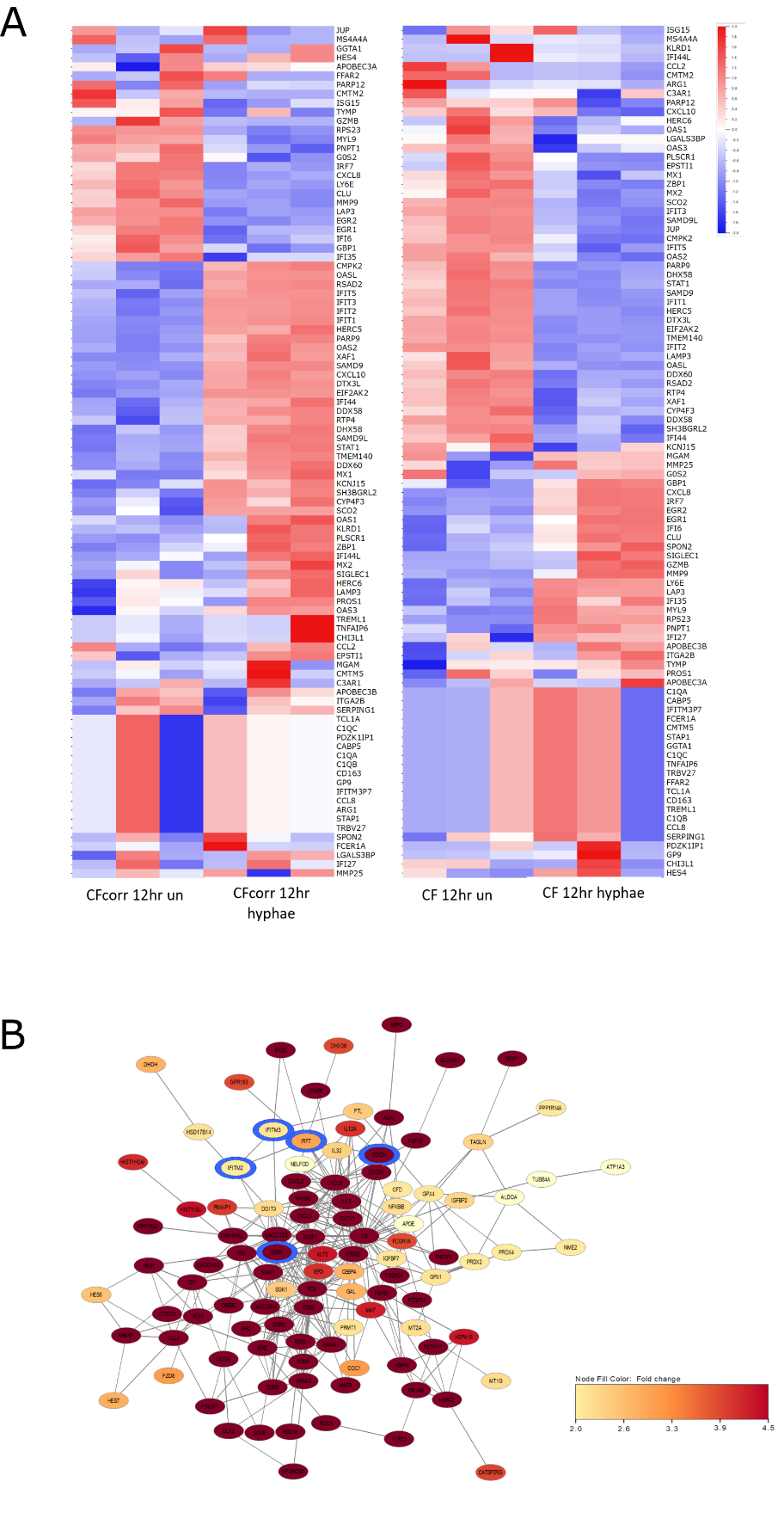
**

**Supplementary Figure 2:** Differentially expressed ISGs of heat killed conidia infected CF and CF corrected BECs after 12 hours (data represents 3 experimental replicates). CF and CF corrected BECs were infected with Af heat killed conidia (MOI=8) for 12 hours, RNA was isolated and sent for bulk RNA sequencing. (A) Heatmap was organised by hierarchical clustering based on mean gene expression and includes genes from a list of 486 ISGs. Each column represents a sample (n=3), and each row represents a gene (padj<0.05), significance calculated by ANOVA. (B) Venn diagram of differentially expressed ISGs compared to unstimulated control of named condition representing overlap of ISG signature in CF and CF corrected BECs. Volcano plots representing differentially expressed ISGs for (C) CF corrected and (D) CF cells infected by Af heat killed conidia for 12 hours, determined by student’s t-test padj<0.05 and fold change >2.

**
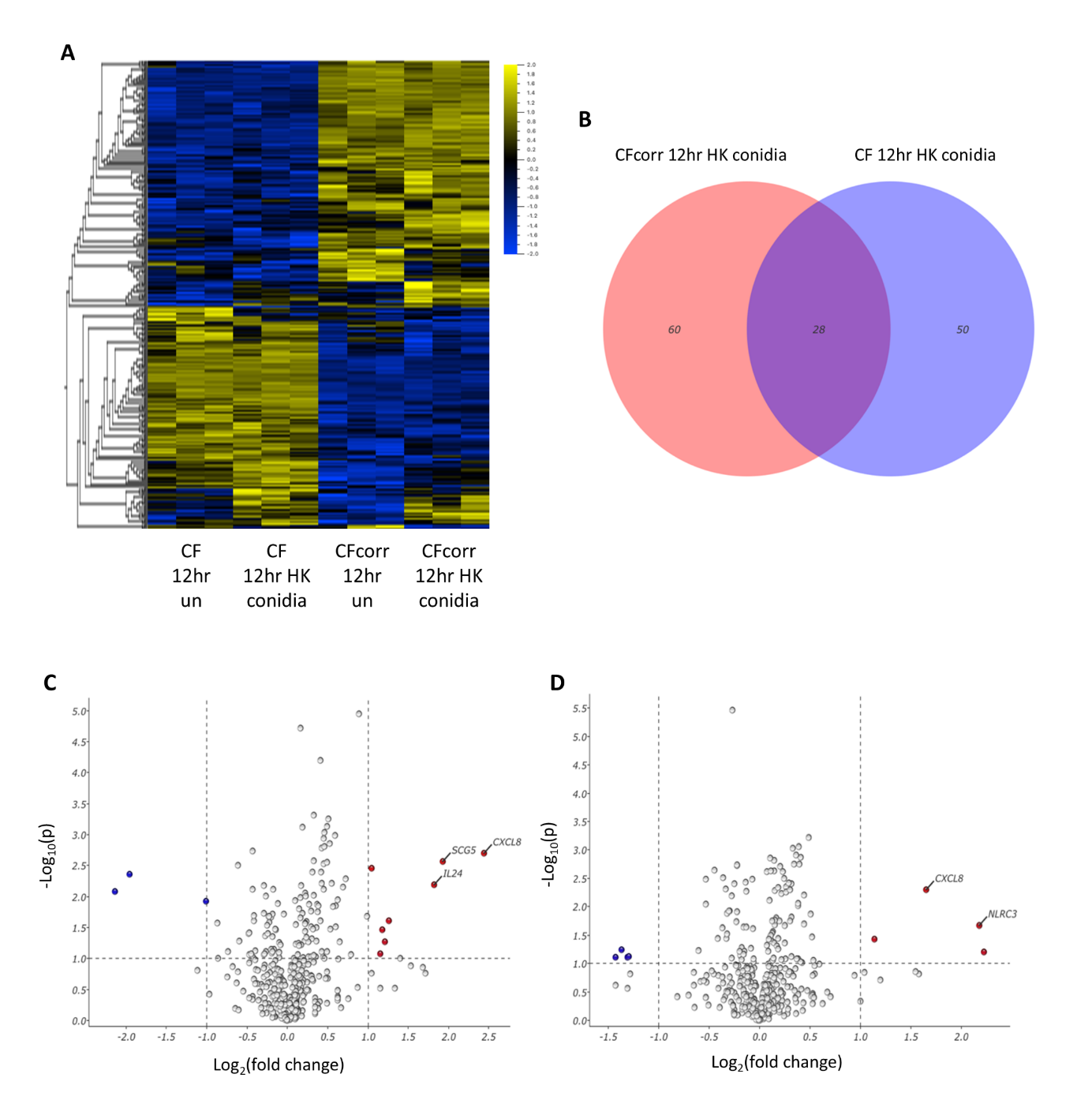
**

**Supplementary Figure 3:** IFNβ and IFN𝝀1 expression by CF and CF corrected BECs after infection with Af fixed hyphae and heat killed conidia (Data represents 3 experimental replicates). CF and CF corrected BECs were stimulated with poly(I:C) (100ug/ml) and infected with fixed Af hyphae (MOI=8) and heat killed Af conidia (MOI=8) for 3, 12 and 24 hours. RNA was isolated from washed cells an RT-PCR was carried out to assess expression of (A) IFNβ, (B) IFN 𝝀1 and (C) IL-8.

**
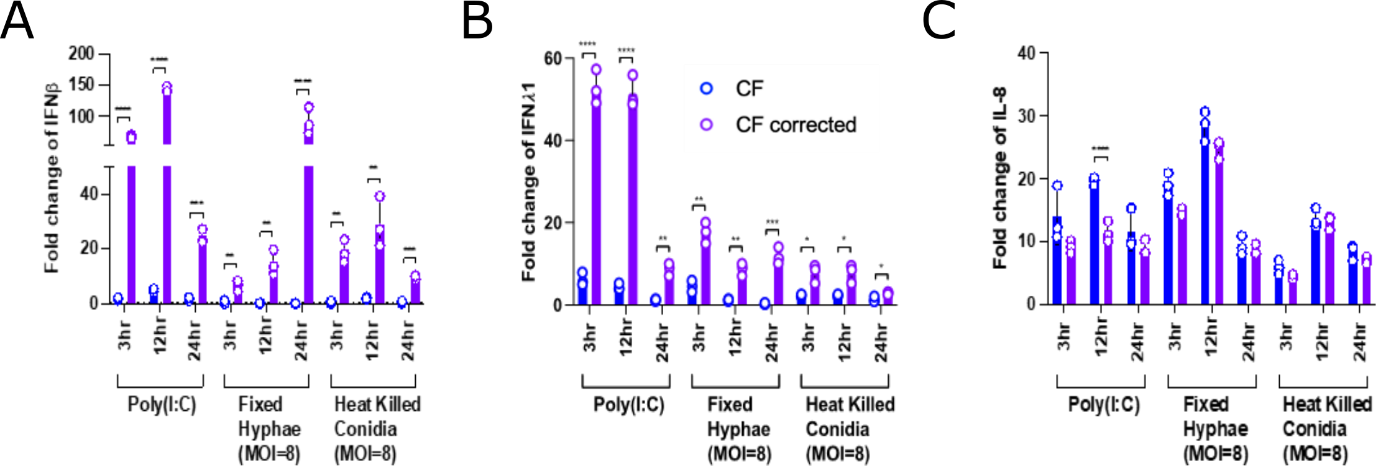
**

**Supplementary Figure 4**: Differentially expressed ISGs of poly(I:C) stimulated CF and CF corrected BECs after 24 hours (data represents 3 experimental replicates). CF and CF corrected BECs were stimulated with poly(I:C) (100μg/ml) for 24 hours, RNA was isolated and sent for bulk RNA sequencing. (A) Heatmap was organised by hierarchical clustering based on mean gene expression and only includes genes from a list of 486 ISGs. Each column represents a sample (n=3), and each row represents a gene (padj<0.05). Significance was calculated using ANOVA. (B) Venn diagram of differentially expressed ISGs compared to unstimulated control of named condition representing overlap of ISG signature in CF and CF corrected BECs. Volcano plots representing differentially expressed ISGs for (C) CF cells and (D) CF corrected stimulated by poly(I:C) for 24 hours, determined by student’s t-test padj<0.05 and fold change >2.

**
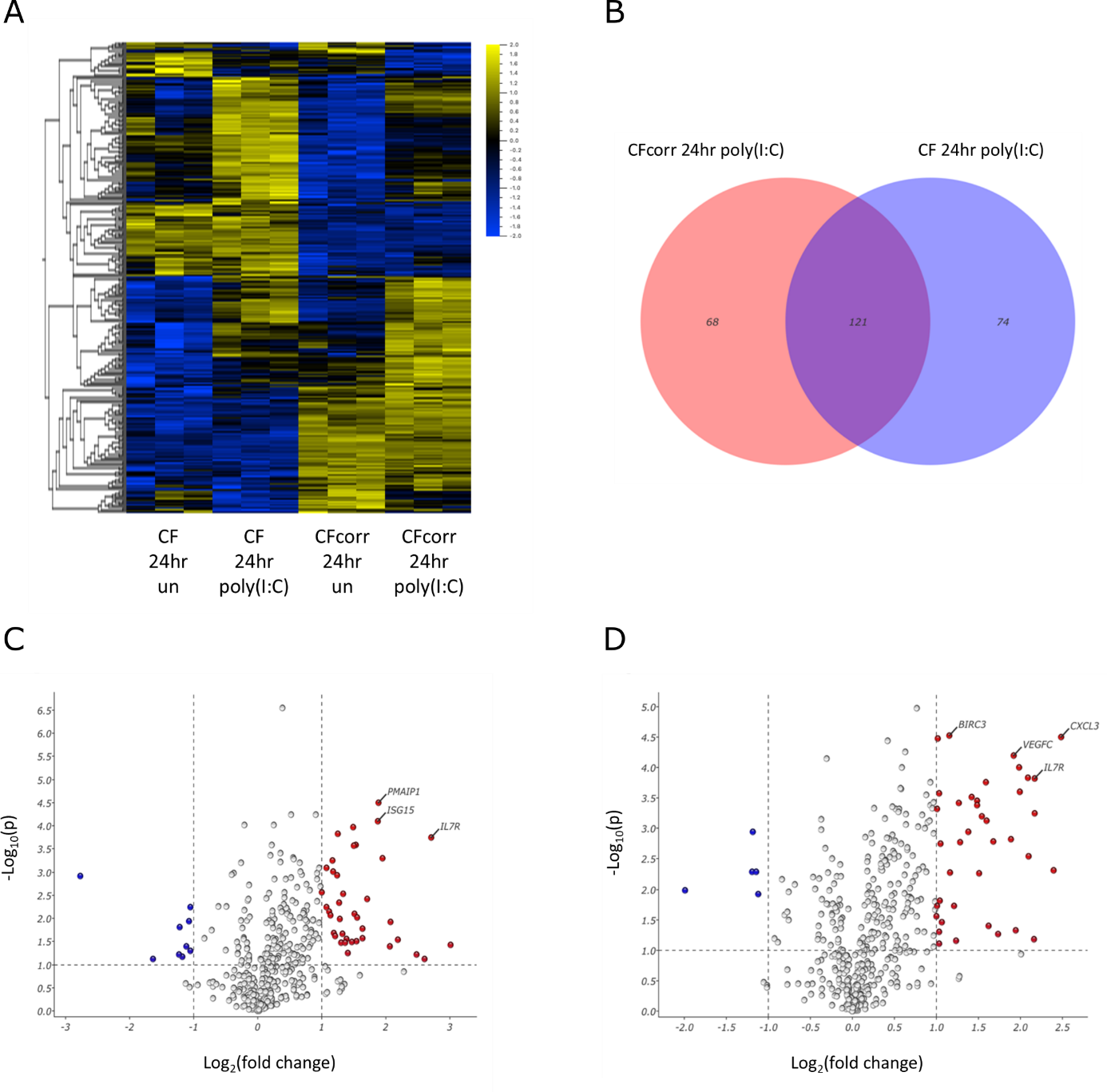
**

**Supplementary Figure 5:** Network analysis of Af heat killed conidia infected CF BECs after 12 hours with and without CFTR modulator treatment (data represents 3 experimental replicates). Network visualisation of significant immunity-associated proteins (padj<0.05 and fold change >2), after 12 hours of heat killed conidia infection in (A) CF BECs and (B) CF BECs with CFTR modulator treatment. The nodes indicate genes, and the colour represents fold change. Functional enrichment analysis highlights the nodes involved in the type I IFN response (blue) (Cytoscape, stringApp).

**
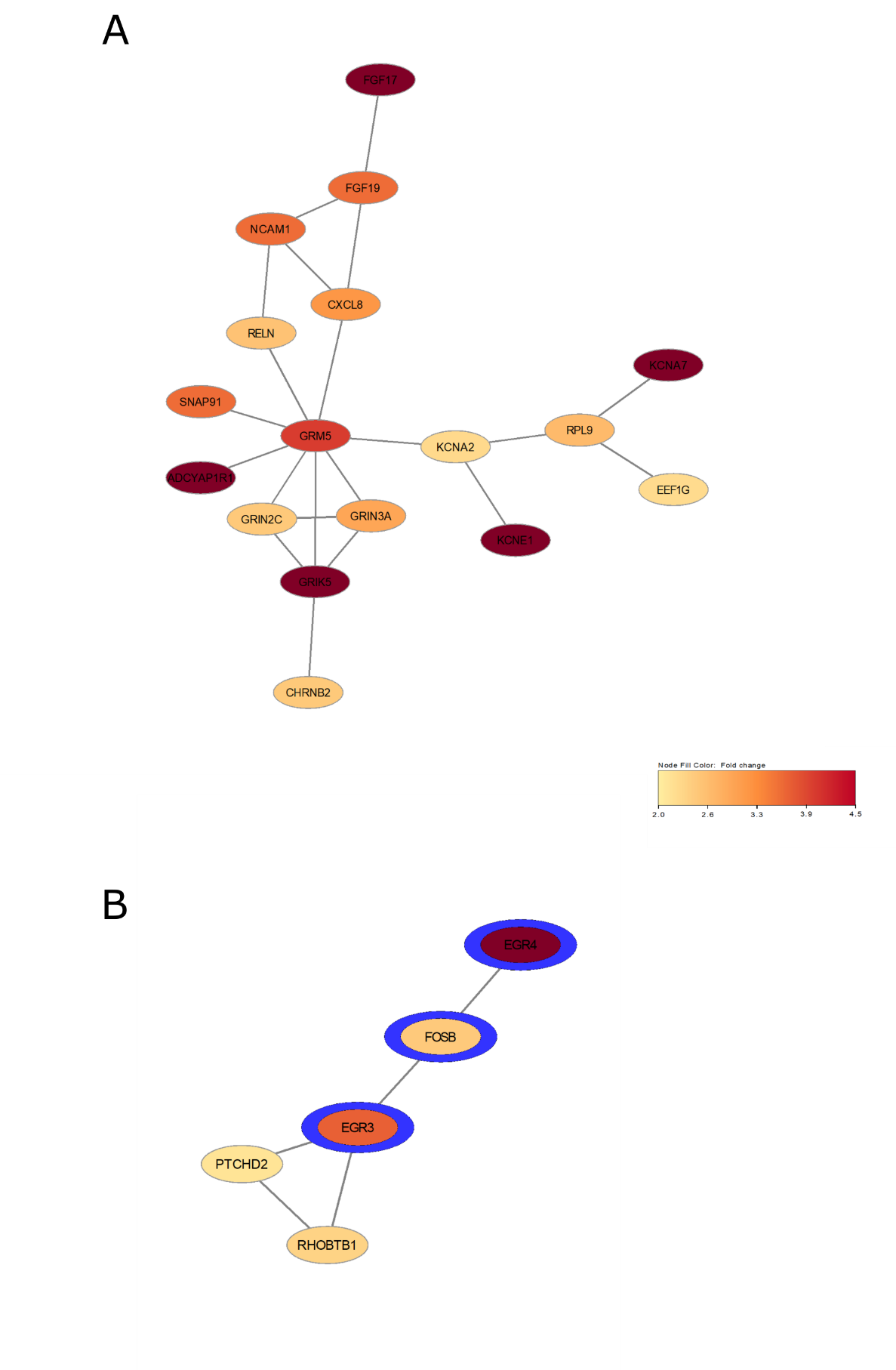
**
