# Supplementary material for: CFTR modulators partially restore the epithelial interferome in Aspergillus infection to improve clinical outcome
Source: eBioMedicine. 2026 Jan 31;124:106131. doi: 10.1016/j.ebiom.2026.106131 (PMC12878693; doi:10.1016/j.ebiom.2026.106131)
Supplement: ICLAC_cell line_completed [file mmc2.pdf]

Manuscript or Grant Information

Title or Manuscript/Grant ID:	EBIOM-D-25-02828R1
Cell Lines used:	CFBE41o- and CFBE41o- 4.7
Cell Lines used with Quality Concerns:	n/a

Indicate "Yes" or "No" for each question below and further comments in the next page. Add new pages, if necessary.

**Cell line designation** / **Reporting Requirements**					
The RRID for the cell line is listed?[1]	✓				
The cell line is known to be problematic?[2]	no				
Authentication testing was performed?[3]	no				
Human cell lines: STR profile is available with the manuscript/grant application?[3]	yes				
Mycoplasma testing has been performed?[4]	yes				
The source for the cell line is listed?[5]	yes				
Sufficient information is given to replicate experiments using the cell line?[6]	yes				

[1] The Resource Identification Initiative (RRID) is meant to help researchers cite the important resources used in scientific papers (See Recommendation #1).
[2] See the ICLAC website for a register of known misidentified cell lines (See Recommendation #2 on Page 1 of this document).
[3] The authentication test method and results should be listed in the manuscript/project (See Recommendation #3 on Page 1 of this document).
[4] The mycoplasma test method and results should be listed in the manuscript/project.
[5] The catalogue number should be included if obtained from a cell line repository. See also Recommendation #4 on Page 1 of this document.
[6] See Recommendation #5 on Page 1 of this document.
